# P02.86. A new method for quantifying the needling component of acupuncture treatments

**DOI:** 10.1186/1472-6882-12-S1-P142

**Published:** 2012-06-12

**Authors:** R Davis, D Churchill, G Badger, J Dunn, H Langevin

**Affiliations:** 1 Stromatec, Inc., Burlington, USA; 2 Dept of Medical Biostatistics, University of Vermont, Burlington, USA; 3 New England School of Acupuncture, Newton, USA; 4 Dept of Neurology, Universtiy of Vermont College of Medicine, Burlington, USA

## Purpose

The highly variable nature of acupuncture needling creates challenges to systematic research. The goal of this study was to test the feasibility of quantifying acupuncture needle manipulation using motion and force measurements. We hypothesized that distinct needling styles and techniques would produce different needle motion and force patterns that could be quantified and differentiated from each other.

## Methods

A new needling sensor tool (Acusensor) was used to record needling in real time as performed by six New England School of Acupuncture (NESA) faculty from the “Chinese Acupuncture” (Style 1) and “Japanese Acupuncture” (Style 2) programs (three from each). Each faculty expert needled twelve points (six bilateral locations) in twelve healthy human subjects using both tonification (Technique 1) and dispersal (Technique 2). Parameters calculated from the raw needling data were displacement amplitude, displacement frequency, rotation amplitude, rotation frequency, force amplitude, and torque amplitude.

## Results

Data analysis revealed significant differences in the amplitude of both displacement and rotation between needling performed by faculty from two different acupuncture styles. We also found significant overall differences in the frequency of displacement between tonification and dispersal that were not dependent of the style of acupuncture being performed. The relationships between displacement and rotation frequencies, as well as between displacement and force amplitudes, showed considerable variability across individual acupuncturists and subjects.

## Conclusion

Needling motion and force parameters can be quantified in a treatment-like setting. Needling data can subsequently be analyzed, providing an objective method for characterizing needling in acupuncture basic and clinical research.

